# IgE and IgG Antibodies as Regulators of Mast Cell and Basophil Functions in Food Allergy

**DOI:** 10.3389/fimmu.2020.603050

**Published:** 2020-12-11

**Authors:** Cynthia Kanagaratham, Yasmeen S. El Ansari, Owen L. Lewis, Hans C. Oettgen

**Affiliations:** ^1^ Division of Immunology, Department of Pediatrics, Boston Children’s Hospital, Boston, MA, United States; ^2^ Department of Pediatrics, Harvard Medical School, Boston, MA, United States; ^3^ Institute of Laboratory Medicine, Philipps University Marburg, Marburg, Germany

**Keywords:** mast cells, IgG, IgE, food allergy, Fc receptor, oral immunotherapy, basophil activation test

## Abstract

Food allergy is a major health issue, affecting the lives of 8% of U.S. children and their families. There is an urgent need to identify the environmental and endogenous signals that induce and sustain allergic responses to ingested allergens. Acute reactions to foods are triggered by the activation of mast cells and basophils, both of which release inflammatory mediators that lead to a range of clinical manifestations, including gastrointestinal, cutaneous, and respiratory reactions as well as systemic anaphylaxis. Both of these innate effector cell types express the high affinity IgE receptor, FcϵRI, on their surface and are armed for adaptive antigen recognition by very-tightly bound IgE antibodies which, when cross-linked by polyvalent allergen, trigger degranulation. These cells also express inhibitory receptors, including the IgG Fc receptor, FcγRIIb, that suppress their IgE-mediated activation. Recent studies have shown that natural resolution of food allergies is associated with increasing food-specific IgG levels. Furthermore, oral immunotherapy, the sequential administration of incrementally increasing doses of food allergen, is accompanied by the strong induction of allergen-specific IgG antibodies in both human subjects and murine models. These can deliver inhibitory signals *via* FcγRIIb that block IgE-induced immediate food reactions. In addition to their role in mediating immediate hypersensitivity reactions, mast cells and basophils serve separate but critical functions as adjuvants for type 2 immunity in food allergy. Mast cells and basophils, activated by IgE, are key sources of IL-4 that tilts the immune balance away from tolerance and towards type 2 immunity by promoting the induction of Th2 cells along with the innate effectors of type 2 immunity, ILC2s, while suppressing the development of regulatory T cells and driving their subversion to a pathogenic pro-Th2 phenotype. This adjuvant effect of mast cells and basophils is suppressed when inhibitory signals are delivered by IgG antibodies signaling *via* FcγRIIb. This review summarizes current understanding of the immunoregulatory effects of mast cells and basophils and how these functions are modulated by IgE and IgG antibodies. Understanding these pathways could provide important insights into innovative strategies for preventing and/or reversing food allergy in patients.

## Introduction

Global surveillance by the World Allergy Organization, shows that the prevalence of food allergies has been rising over the last decade in both developed and developing countries (1). This increase has been considered as the “second wave” of the allergy epidemic, following the “first wave” that was driven by allergic respiratory illnesses. In the United States alone, food allergies affect approximately 8% of children and 2%–3% of adults (2). The most common food allergens in the US are peanut, cow’s milk, hen’s egg, tree nut, soy, fish, wheat, and shellfish (3).

Food allergies are hypersensitivity reactions that can be mediated by a wide range of humoral and cellular mechanisms. IgE-mediated food allergy is the most common and will be the focus of this review. It occurs in individuals who produce food-specific IgE antibodies. These subjects are often referred to as “sensitized”. These IgE antibodies are bound to the innate granulocytic effector cells of anaphylaxis, mast cells and basophils. Upon interaction with allergen and cell-bound IgE, the granule contents of these cells are released and, along with prostaglandin and leukotriene mediators rapidly produced by the same cells, act on a range of target tissues to trigger immediate physiologic responses (4). In the vasculature, these mediators cause dilation of blood vessels and increased plasma leak which manifest locally in tissues as hives and angioedema (including laryngeal edema) and systemically as hypovolemic shock (4). The mediators additionally cause smooth muscle constriction, leading to bronchospasm, vomiting, and diarrhea, and also bind to neuronal receptors triggering pain and itch (4). When multiple organ systems are involved, the reaction is designated systemic anaphylaxis (3). Acute reactions often resolve within the first few hours; however, some patients experience recurrence of symptoms 8–12 h following the first reaction (biphasic reactions) (5).

The mechanisms of immunological priming leading to IgE production in food allergic subjects are unclear. Some patients have prior histories of ingestion, suggesting immunological sensitization *via* the gut. However, many children experience adverse reactions following their initial ingestion of a food, suggesting alternate routes of immune priming. Emerging evidence suggests that sensitization can occur following cutaneous contact, especially in the setting of a disrupted skin barrier, as occurs in atopic dermatitis (6, 7).

Our understanding of the pathways of immunological sensitization, effector cell activation and regulation of IgE-mediated food allergy has grown rapidly since just over 50 years ago when reagin, the fraction of serum responsible for transferring skin test responsiveness from an allergic individual to a naïve recipient, was identified as IgE. The factors regulating IgE-mediated food allergy have been of great interest with a particular emphasis in the role of regulatory T cells (Tregs) in constraining both the emergence of food allergen-specific T helper cells and the production of allergen-specific IgE. However, in recent years, the ability of mast cells and basophils to exert adjuvant functions in immune sensitization to allergens and of IgG antibodies to block IgE-mediated food allergy has been recognized and the role of the inhibitory IgG receptor, FcγRIIb, in potently inhibiting food allergies has really come into focus. In the first part of this review, we briefly discussed the mechanisms, pathophysiology and key players in the disease. In the second part, we cover the evidence for a regulatory functions of mast cells, basophils, IgE and IgG and how they may be targeted clinically to counter food allergy.

## Mechanisms, Pathophysiology, and Treatment of Food Allergies

### Food Allergy, a Breakdown of Oral Tolerance

Our ability to maintain systemic unresponsiveness to orally ingested antigens is an active process occurring in gut-associated lymphoid tissues. Food antigens can cross the epithelial barrier following damage to the epithelium, through specialized intraepithelial passages, or *via* sampling by antigen presenting cells (APCs) (8). Oral exposure promotes the development of Foxp3^+^ Tregs, including RORγt^+^ Tregs that are induced by microbial signals in a Myd88-dependent manner (9–11). These prevent the development of allergen-specific IgE specialized CD103^+^ dendritic cells in the gut, *via* a process involving TGF-β and retinoic acid, promote the differentiation of naïve T cells into Tregs (12). A break in tolerance can occur when the cytokine environment in the intestine favors the emergence of effector T helper 2 (Th2) cells and/or the reprogramming of Tregs to a pathogenic phenotype. Cytokines produced by gut epithelial cells, including IL-25, IL-33 and thymic stromal lymphopoietin (TSLP), may be particularly important drivers of this shift away from tolerance. IL-25 expression has been shown to be high in the small intestine in mouse models of food allergy, and overexpression of IL-25 increases Th2 cytokine production by type 2 innate lymphoid cells (ILC2s) and amplifies allergic responses. Conversely, lack of IL-25 is protective (13, 14). TSLP is similarly expressed in gastrointestinal epithelial cells in the setting of food allergy. Khodoun and colleagues demonstrated that monoclonal antibodies against IL-25, IL-33, or TSLP could each individually prevent the development of food allergy induced by oral gavage with egg white and medium chain triglycerides in mice (15). IL-33 produced by intestinal epithelial cells leads to an increase in OX40L expression on CD103^+^ dendritic cells, which skews the immune profile towards Th2 (16, 17). IL-33 produced by keratinocytes following epicutaneous allergen exposure to damaged skin in mice, as can occur in atopic dermatitis, has been shown to promote activation of ILC2s, Th2 skewing, intestinal mast cell expansion and susceptibility to anaphylaxis (18). By increasing the production of IL-4 by ILC2s, IL-33 can also suppress the development of Tregs (19). Researchers at Stanford have shown that etokimab (anti-IL-33) (NCT02920021) can protect against reactions to oral peanut challenge, reduce allergen-specific IgE, and decrease the levels of cytokines associated with food allergies such as IL-9 and IL-13 (20).

Some of the pro-sensitizing effects for food allergens are exerted by these same cytokines in the skin. In a mouse model of atopic dermatitis driven by the cutaneous application of ovalbumin (OVA) along with the Vitamin D analogue MC903, Noti, and colleagues observed that TSLP produced by keratinocytes promotes basophil expansion and the induction of a Th2 response (driven by basophil-derived IL-4) with food allergy (21, 22). Muto and colleagues reported a similar TSLP- and basophil-driven food allergy response in mice epicutaneously sensitized in the presence of SDS (23). Leyva-Castillo and colleagues have shown that exposure to food allergens *via* disrupted skin barrier has also been shown to promote mast cell expansion at a distance in the small intestine in a manner dependent on the induction of intestinal ILC2s by keratinocyte-derived IL-33 along with intestinal tuft cell-derived IL-25 (18).

### Gastrointestinal Mast Cell Expansion in Food Allergy: IL-4, IL-9, and MMC9

In addition to IL-25 and IL-33, the Th2 cytokines IL-4 and IL-9 have been shown to play an important role in food allergy development. IL-9 is produced both by Th2 cells and by a subset of mast cells, IL-9-producing mucosal mast cells (MMC9). IL-9 promotes mast cell growth and lack of IL-9 prevents the induction of food allergy in mouse models (24, 25). Transgenic overexpression of IL-9 promotes the food allergy response by increasing mast cell number in the small intestine and enhancing gut permeability (24, 25). Th2 cells contribute to the development of MMC9 that provide a positive feedback loop for the expansion of mast cell numbers following oral challenge (26). IL-4, in addition to its role in promoting Th2 responses and driving IgE isotype switching in B cells, is also needed to support intestinal mast cell expansion following food allergen ingestion (27). This cytokine induces the antiapoptotic genes, Bcl-2 and Bcl-X(L), in mast cells in a STAT6-dependent manner (27). Competitive reconstitution studies using wild type mouse mast cells mixed with mast cells lacking the IL-4 receptor α-chain have revealed a strong competitive advantage of IL-4 receptor-bearing cells. Hogan and colleagues have recently demonstrated that IL-4, signaling *via* IL-4Rα induces MMC9 in a BATF-dependent mechanism, nicely linking the observed roles of IL-9 and IL-4 in mast cell homeostasis (28). IL-4 signals can subvert Tregs from their normally suppressive phenotype to one in which they express the Th2 transcription factor GATA-3 and Th2 cytokines, including IL-4, and thereby drive the disease phenotype (29). Investigators at Stanford and elsewhere are conducting trials exploring the potential of dupilumab (anti-IL-4Rα monoclonal antibody that prevents the binding of IL-4 and IL-13 to its receptor) in combination with anti-IgE (NCT03679676) during peanut oral immunotherapy (OIT).

### Treatments for Food Allergy

Currently, there are no curative treatments for food allergies. The standard approach is education of individuals and families regarding strategies of allergen avoidance. While this is effective in preventing potentially serious reactions, it can significantly impact the quality of life of the patient and their family members. Avoidance paradoxically deprives these patients of one of the best-known paths to achieving tolerance to an antigen, namely ingestion. Furthermore, broad avoidance diets can result in nutritional deficiencies in multi-sensitized children (30, 31). Some children will eventually experience natural resolution of their allergy. Still, while this tends to occur for some foods, like egg and dairy, it is rare for others, like peanuts and tree nuts. In addition to practicing allergen avoidance, patients diagnosed with food allergies, must be presented with effective treatment plans on how to manage their reactions in case of accidental exposure. Mild reactions to foods, such as itching and hives, can be treated with anti-histamines, such as diphenhydramine and cetirizine. Though anti-histamines can alleviate the symptoms associated with allergy, they do not hinder the progression of the disease or reverse the disease-associated immune profile. Systemic anaphylaxis, the severe, life threatening reaction, presents as difficulty breathing or swallowing, and is treated immediately with epinephrine. Immunotherapy, which involves exposure to increasing doses of the allergen over a period of time, is the only disease-modifying treatment. Immunotherapy can be administered *via* several routes, including sublingual (SLIT), epicutaneous (EPIT) and oral (OIT) (32). Peanut powder (Palforzia^®^) OIT is the only current FDA-approved treatment for immunotherapy. A major limitation of OIT is that the food unresponsive state that is achieved is transient and maintaining it requires continued regular ingestion of the food (33). Adjunctive anti-cytokine treatments that might enhance safety as well as durability of immunotherapy are actively being explored (20, 34).

## Key Players Involved in Food Allergy: Mast Cells And Basophils, lgE, and lgE Receptors

### Mast Cells and Basophils: Effectors of Immediate Hypersensitivity

Mast cells and basophils arise from CD34^+^ hematopoietic progenitor cells. Mast cells are long-lived tissue resident cells that differentiate locally from bloodborne progenitors (35). They are often found in vascularized sites that are exposed to the external environment and microbiome, such as the mucosa of the gastrointestinal and respiratory tracts (36). Basophils are short-lived cells that mature in the bone marrow, enter the circulation and can either be activated intravascularly or traffic to sites of inflammation to exert their functions (37–39). Because of their anatomic localization in barrier tissues, mast cells are likely to be one of the first cell types to encounter and respond to pathogens, making them important effector cells of the innate immune response. Several groups have identified their protective roles in bacterial infection and they are also critical in the immune response to parasites (36, 40). They express pathogen recognition receptors and can release anti-microbial peptides upon activation. Mast cell granule proteases play an important role in detoxifying insect, scorpion, and reptile venoms (41–43). Like mast cells, basophils have been demonstrated to play key roles in host defense not only in the setting of Th2 responses, but also in the inflammatory reactions leading to helminth expulsion or tissue encystment and in resistance to ticks (44–48).

In addition to acting as effector cells of innate immunity, mast cells and basophils live at the interface of innate and adaptive immunity. Since they express Fc receptors, for IgE and IgG, they are armed with the adaptive immune capability to recognize specific antigens and participate in recall responses. They also regulate the emergence of adaptive responses. Granule components such as histamine can regulate T cell immunity and the antibody response, and mast cells and basophils are major producers of IL-4 and IL-13 (49–52). These two cytokines promote both Th2 cell differentiation and isotype switching of B cells to produce IgE.

The classic trigger for mast cell and basophil activation is through the IgE-mediated crosslinking the IgE receptor, FcϵRI. This activation results in the degranulation of the cells, releasing preformed mediators (such as histamine, neutral proteases, and TNF-α), *de novo* synthesis of pro-inflammatory lipid mediators, and production of growth factors, cytokines, and chemokines (53, 54).

### FcϵRI and Its Downstream Signaling Pathways

IgE is the least abundant antibody in circulation. Its concentration in plasma is roughly 10^5^ times less (100 ng/ml vs 10 mg/ml) than that of IgG. In addition to being present at such low concentrations, IgE has a notably short half-life, less than 1 day, while that of IgG is around 3 weeks (55). However, most of the IgE in the body is found in a cell-bound state, owing to the incredibly high affinity for IgE by FcϵRI (K_d_ 1x10^-9^mol/L). IgE effectively remains permanently attached to FcϵRI until internalized, which makes the tissue half-life of IgE to likely be on the order of weeks to months (55, 56).

There exist two isoforms of the high affinity IgE receptor, FcϵRI. The tetrameric αβγ_2_ form is found on mast cells and basophils. The trimeric αγ_2_ form, though less abundant than the tetrameric, is present on eosinophils, platelets, monocytes, and dendritic cells. The alpha chain has the ligand binding site, with two Ig-like domains that bind IgE. The β chain contains four transmembrane spanning regions and it amplifies the signal generated by the γ subunit. The γ chains are dimeric disulfide-linked transmembrane proteins. The β and γ chains contain immunoreceptor tyrosine-based activation motifs (ITAMs). An ITAM is a conserved sequence containing tyrosine separated from a leucine or isoleucine by two amino acids (YxxL/I). The ITAM tyrosine is a substrate for phosphorylation by signaling kinases such as Lyn and Syk. Two ITAMs are often found together separated by 6 to 8 amino acids (YxxL/I x(6-8) YxxL/I).

**Figure 1:**
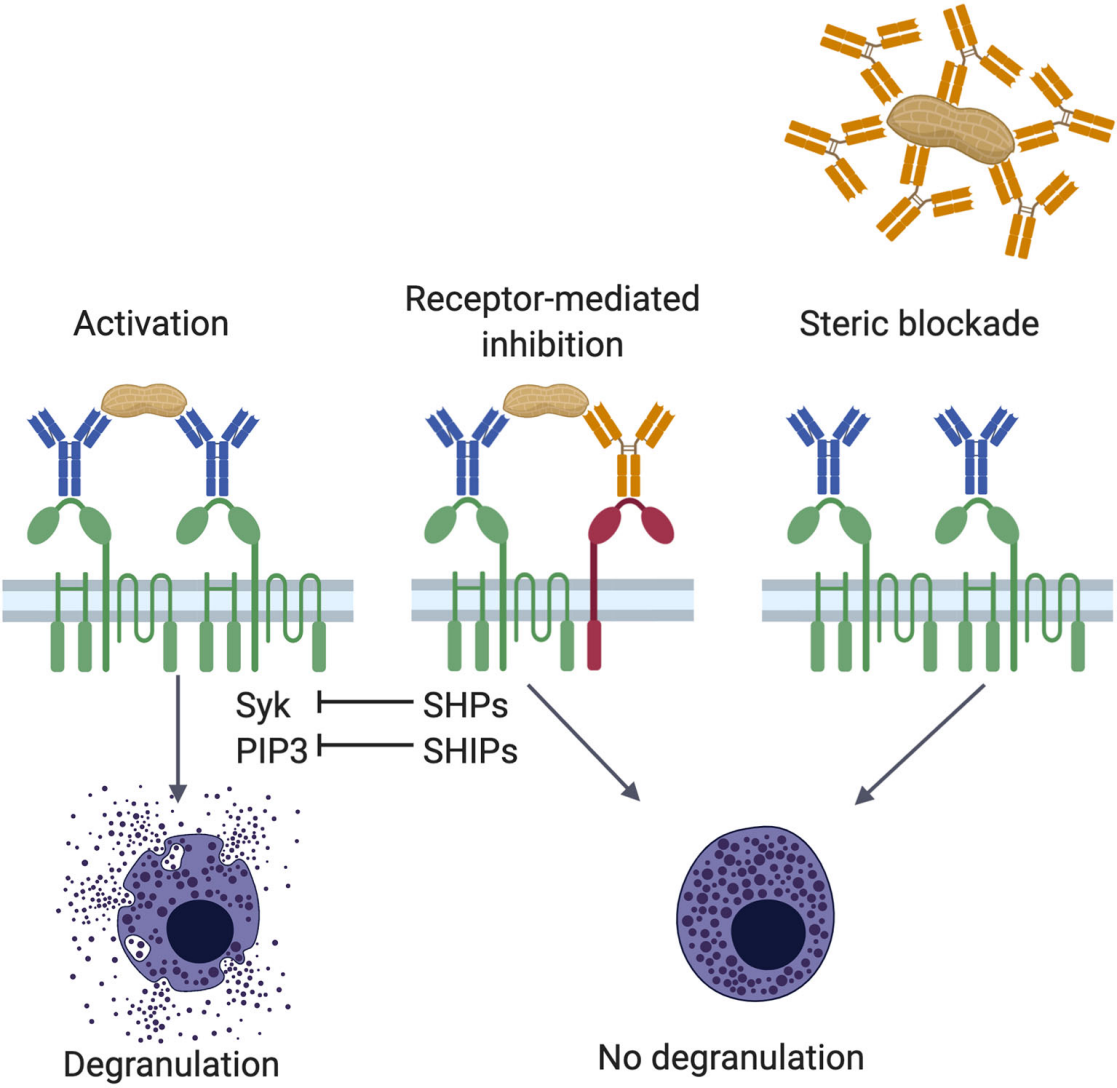
****Mechanisms of IgG mediated inhibition. Antigen encounter by neighboring FcϵRI (green)-bound IgE (blue) on the surface of mast cells or basophils induces receptor crosslinking, phosphorylation of cytosolic immunoreceptor tyrosine-based *activation* motif (ITAM) sequences in the tetraspanning β- and disulfide-linked γ-chain dimers, and activation of various signaling pathways involving protein tyrosine kinases, such as Syk, and inositol intermediates, including PIP_3_ (left panel). These positive signals culminate in the degranulation of the cell. Allergen specific IgG antibodies (orange) counter the effects of IgE in two ways, receptor-mediated inhibition (center panel) and steric blockade (right panel). In receptor-mediated inhibition, when polyvalent allergens are simultaneously engaged by FcϵRI-bound IgE and FcγRIIb (red)-bound IgG, crosslinking of the two receptors leads to phosphorylation of FcγRIIb cytosolic immunoreceptor tyrosine-based *inhibition* motifs (ITIMs). These recruit protein tyrosine phosphatases and inositol phosphatases, such as SHPs and SHIPs, respectively. Phosphatases can neutralize phosphoprotein (such as Syk) and phospholipid (such as PIP_3_) signaling intermediates induced by FcϵRI activation. In steric blockade, IgG antibodies bind the allergen before it reaches receptor-bound IgE. By masking IgE binding epitopes, these blocking IgG antibodies inhibit interaction with IgE and thereby prevent FcϵRI-mediated mast cell activation.

When multivalent antigens bind to FcϵRI-bound IgE, proximal FcϵRI aggregate into lipid rafts that are rich in cholesterol, sphingolipids, protein tyrosine kinases (PTKs), and GPI-anchored proteins. Recognition of one antigen molecule by Fab sites on two different IgE molecules bound to neighboring FcϵRI, results in receptor crosslinking and transphosphorylation of the ITAMs. Antigen recognition mediates a rapid cascade of signaling events that induce the activation of PTKs of the SRC and TEC families (57–59). This activation leads to the release of preformed, rapidly formed, and newly synthesized mediators from the effector cells (60) (see **Figure 1**).

Tyrosine residues on ITAMs on β and γ chains are phosphorylated by receptor-associated Lyn tyrosine kinase found constitutively associated with the β chain. Phosphorylation of tyrosine residues in the ITAMs provides docking sites for Src homology 2 (SH2) domain-containing kinases, such as Syk PTK to the γ chains and recruits additional Lyn PTK. Syk is phosphorylated by Lyn and activated by conformational changes (61, 62). Phosphorylated Syk subsequently phosphorylates adapter molecules LAT1, LAT2, SLP-76, Grb2, and VAV at tyrosine sites leading to the formation of supramolecular plasma membrane-localized signaling complexes. These signaling events lead to the activation of other signalling pathways such as PI3K, PLC-γ, RAS/ERK, JNK, p38, and AKT. They also lead to the immediate exocytosis of granules and their pre-formed mediators, rapid synthesis of prostaglandins and leukotrienes and eventual transcription of cytokines genes, such as IL-4, IL-6, IL-10, and TNF-α (60).

In addition to driving the allergic effector functions of mast cells and basophils, there is evidence that some IgE antibodies can provide survival signals to mast cells even in the absence of allergen. These have been designated “cytokinergic” IgEs by Kawakami and colleagues (63). In the absence of the survival cytokines necessary for mast cells, such as SCF, cytokinergic IgE antibodies can provide a survival-enhancing effect (64, 65). Furthermore, they have been shown to induce cytokine production, calcium flux, histamine release, and leukotriene synthesis (66–68). Cytokinergic IgE antibodies may act by self-associating or binding to autoantigens in order to aggregate FcϵRIs.

Glycosylation of IgE molecules has been shown to correlate with IgE binding to FcϵRI and for effective signaling upon receptor cross-linking. IgE antibodies are very heavily glycosylated when compared with other immunoglobulin isotypes. Anthony and colleagues demonstrated the functional significance of IgE glycans. They found that one at N394 was obligatory for FcϵRI binding (69). Subsequent comparison of plasma IgE molecules between allergic and non-allergic individuals by mass spectrometry by the same group showed that the “allergic IgE” has more terminal sialylation of its glycans than “non-allergic IgE”. The degree of sialylation of IgE does not affect its binding to FcϵRI but modulates the signaling downstream of the receptor and activation of the cell (70).

### CD23: The Low Affinity Receptor for IgE

**Figure 2:**
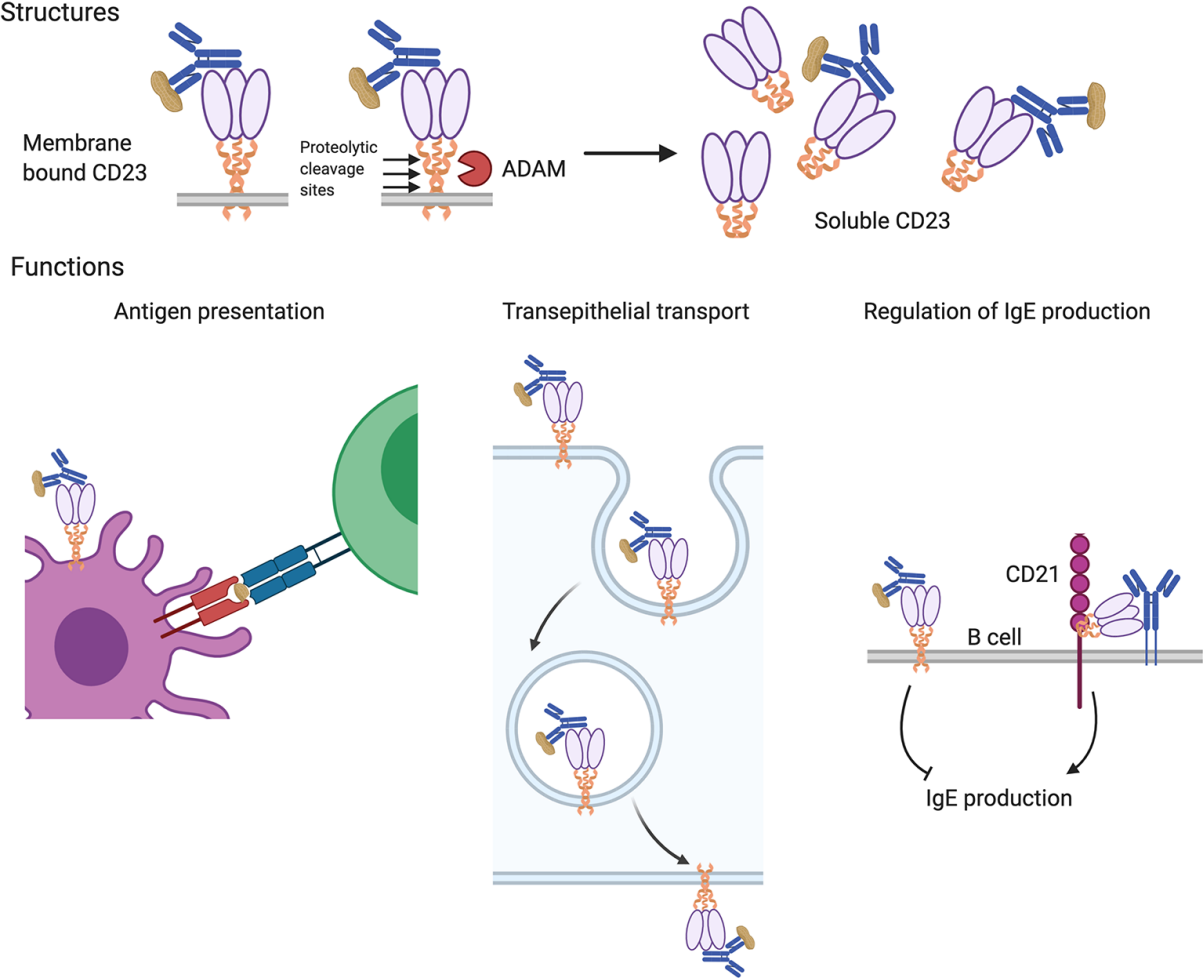
Structures and functions of the low-affinity IgE receptor, CD23. CD23 is expressed as a multimer of subunits consisting of coiled-coil stalks with lectin-family domain heads that bind to IgE (upper panels). Membrane-bound CD23 can be converted to a soluble form that retains IgE-binding ability following cleavage at protease sites by endogenous (ADAM) or allergen (e.g. *Der p 1*) proteases. Various functions have been attributed to CD23 (lower panels). It can facilitate antigen uptake for presentation by B cells and antigen presenting cells to T cells (left panel) and mediate the transport of allergens across polarized epithelium in the gut and airway (center panel). CD23 also regulates IgE production; the transmembrane form on B cells suppresses their production of IgE and the soluble form, *via* interactions with B cell surface CD21 and IgE, enhances IgE production (right panel).

IgE can also bind to and exert its effects through its low affinity receptor CD23 (FcϵRII). CD23 is broadly distributed with expression on B cells, dendritic cells, eosinophils, gastrointestinal and respiratory epithelial cells, and others (71). It is a type II transmembrane protein (N-terminus intracellular) assembled as a multimer with α-helical coiled-coil stalks terminating in C-type lectin heads that bind to IgE (see **Figure 2**) (72). CD23 is the only Fc receptor that is not part of the immunoglobulin superfamily. In addition to binding IgE, it also interacts with the B cell surface protein, CD21, which functions as complement receptor 2 (CR2) and is the binding site and entry point for Epstein-Barr virus in B cells (73). Protease-sensitive sites present in the stalks of CD23 can be cleaved by endogenous proteases such as the metalloproteinase, ADAM10, and by protease allergens including the dust mite protease, *Der p 1*. The released oligomeric CD23 heads are called soluble CD23 (sCD23) and retain their ability to bind IgE.

## lgG Receptors

In addition to FcϵRI, human and mouse mast cells and basophils also express Fcγ receptors (FcγRs) that bind to IgG antibodies. FcγRs belong to the immunoglobulin superfamily and their patterns of expression vary among leukocytes (74–77). The general consensus is that human mast cells and basophils express FcγRIIa and FcγRIIb, while mouse mast cells and basophils express FcγRIIIa and FcγRIIb (78). FcγRIIb is the only inhibitory IgG receptor, and all others are activating. IgG and FcγRs are involved in a number of immune defense mechanisms including toxin neutralization, antibody dependent cell-mediated cytotoxicity, phagocytosis, and cytokine production.

### Activating IgG Receptors: IgG-Mediated Anaphylaxis

The classical paradigm for allergic reactions centers on antigen aggregation of IgE, bound to FcϵRI on mast cells and basophils, leading to their activation. However, even before the discovery of IgE, IgG antibodies had been shown to be able to activate mast cells. In the 1950s, Ovary, Benacerraf and others found that the ability of serum to transfer cutaneous hypersensitivity from one animal to another resided in both a heat-stable gamma-globulin fraction of serum (IgG) and in a heat-labile reagin fraction (79, 80). The relevance of these observations in cutaneous hypersensitivity was confirmed for systemic anaphylaxis more than four decades later when it was reported that active systemic anaphylaxis can be elicited in *i.v*.-challenged mice lacking the Cϵ exons encoding the IgE heavy chain (76, 81–83). Like FcϵRI, crosslinking of activating IgG receptors by immune complexes results in the phosphorylation of ITAMs and a cascade of signaling events (84, 85). While FcϵRI-mediated anaphylaxis is histamine dependent, FcγRIIIa-mediated anaphylaxis involves the release of platelet activating factor from macrophages (86). As demonstrated in mice, IgG-mediated anaphylaxis, unlike IgE-mediated anaphylaxis, occurs predominantly following intravascular antigen exposure and has been shown to require much higher doses of the antigen (86). The existence of IgG-mediated anaphylaxis in humans has not been directly proven and remains somewhat controversial. However, the elicitation of reactions with the physiologic features of anaphylaxis after intravascular administration of agents to which no IgE antibody response can be detected suggests the existence of an IgG pathway. The integration of input signals from activating and inhibitory receptors determines the outcome of the local and systemic inflammatory responses.

### FcγRIIb: The Inhibitory IgG Receptor

FcγRIIb is the only inhibitory IgG receptor and one of its main function is to turn off signals initiated by activating Fc receptors and the B cell receptor (BCR). The importance of IgG-mediated feedback control of humoral immunity by FcγRIIb has long been appreciated (87). Co-aggregation of FcγRIIb with the BCR increases the BCR activation threshold and suppresses B cell-mediated antigen presentation. In the absence of FcγRIIb, the amount of antigen needed to activate B cells is increased, and production of antibodies to T cell-dependent antigens is suppressed (88). FcγRIIb signals can inhibit the maturation of dendritic cells as well as their FcγR-mediated antigen presentation and T cell priming (89, 90). In macrophages, FcγRIIb inhibits FcγR-mediated phagocytosis and release of cytokines (91–93). Overall, FcγRIIb is the most widely expressed FcγR and its presence on various cell types is required for maintenance of peripheral tolerance.

Unlike activating receptors, the aggregation of FcγRIIb alone is of no consequence and binding of IgG or immune complexes solely to FcγRIIb does not inhibit the functions of a resting cell. The inhibitory receptor is expressed on the surface of cells in conjunction with activating receptors, and arming the inhibitory function of FcγRIIb requires an initial licensing signal by an activating receptor. When immunoglobulins on activating and inhibitory receptors recognize independent epitopes in cis on a common antigen, the receptors co-aggregate in lipid rafts for crosslinking to occur (94, 95). FcγRIIb lacks the gamma chain and therefore has no ITAMs. Instead it contains an immunoreceptor tyrosine-based inhibitory motif (ITIM) in its cytosolic domain. ITIMs recruit protein tyrosine phosphatases and inositol phosphatases (96).

ITIMs were first identified in FcγRIIb and subsequent sequence alignments revealed their presence in a large number of inhibitory receptors (96, 97). Following ligand binding and aggregation with an activating receptor, tyrosine residues in these ITIMs are phosphorylated (98, 99). These ITIM phosphotyrosines are docking sites for Src homology 2 (SH2) domain-containing protein tyrosine phosphatases (SHPs) and inositol phosphatases (SHIPs). SHIP-1 dephosphorylates phosphatidylinositol (3, 4, 5)-triphosphate (PIP_3_) to phosphatidylinositol diphosphate (PIP_2_) (see **Figure 1**). This reaction depletes membrane PIP_3_ docking sites for PH-domain-containing signaling intermediates, such as BTK, and consumes the substrate in the formation of IP_3_, which participates in mast cell activation by inducing calcium release from endoplastic reticulum stores. Mast cells deficient in SHIP-1 degranulate at lower concentrations of IgE and antigen, and they also degranulate much more strongly (100). Therefore, SHIP-1, at baseline, raises the threshold for FcϵRI mediated mast cell activation.

It is commonly understood that the inhibitory function of FcγRIIb is mediated by its coaggregation with an activating receptor by the same multivalent antigen. This is described as FcγRIIb-mediated “cis-inhibition”. However, Malbec et al. show that FcγRIIb can also mediate inhibition of activating receptors triggered independently of, and not co-aggregated with, FcγRIIb (101). The authors describe this phenomenon as “trans-inhibition”. Trans-inhibition can decrease the release of β-hexosaminidase, histamine, LTC-4, MIP1-α, and TNF-α from mast cells and basophils, and decrease anaphylaxis. The F(ab)_2_ portion of IgG was not capable of mediating this inhibitory effect, suggesting that the phenotype is dependent on receptor-mediated action of IgG.

### FcγRIIb in Disease

Due to its known roles in inhibiting the activation of immune cells, FcγRIIb has been considered as a potential regulator in various immunological disorders, especially in autoimmune diseases.

Systemic lupus erythematosus (SLE) is a chronic autoimmune disease with autoantibody production affecting many organs including the skin, brain, joints, and kidneys. Abnormal B cell responses to immune complexes containing autoantigens is a feature of SLE. Immune complexes exert their effects *via* activating FcγRs, and impaired FcγRIIb function has been shown to be involved in the disease pathogenesis (102). In rheumatoid arthritis (RA), autoantibodies mediate the destruction of the synovial membrane. Variants in FcγRIIb have been shown to be associated with RA (103). In a collagen-induced arthritis mouse model of RA, lack of FcγRIIb increases the disease score, cartilage destruction, and concentration of collagen specific IgG. In patients with idiopathic thrombocytopenic purpura (ITP), the immune recognition of autoantigens on platelets triggers their destruction. The same variant form of FcγRIIb identified in patients with RA has been shown to be associated with ITP (104). In a mouse model of multiple sclerosis, lack of FcγRIIb is associated with increase in disease score and more activation of myelin-specific T cells. Administration of intravenous immunoglobulin (IVIG) has been successfully used to treat patients with autoimmune diseases during acute flares. IVIG induces the expression of FcγRIIb on blood basophils, monocytes, and eosinophils (105). In contrast, lack of FcγRIIb, or variants of the protein that decrease its function may confer an advantage for fighting infections (106). For example, FcγRIIb deficiency is associated with resistance to streptococcal infections in mice (93, 107). Variants of FcγRIIb associated with SLE (FCGR2B^T232^) have been shown to be more prevalent in areas where malaria is endemic and may confer protection against the infection (108).

## Regulation of Immune Responses by lgE And lgG Antibodies Acting on Mast Cells and Basophils

The opposing effects of IgE antibodies, triggering activating input *via* FcϵRI, and IgG antibodies, sending ITIM-mediated inhibitory signals *via* FcγRIIb, are critical in controlling the acute effector functions of mast cells and basophils. These are the pathways that respectively drive immediate hypersensitivity reactions in food allergy, including anaphylaxis, and suppress acute reactions in subjects who have developed food-specific IgG responses. However, the opposing roles of IgE and IgG in food allergy extend significantly beyond these direct effects on effector cell activation, with immunoregulatory influences ranging from modulation of IgE receptor density and signaling thresholds in effector cells, to the IL-4-driven priming and maintenance of effective Th2 responses to food allergens and corresponding suppression and/or pathogenic reprogramming of Tregs. These immediate and downstream functions of food allergen-specific IgE and IgG antibodies extend the rationale for IgE blockade by omalizumab during OIT and form the mechanistic basis for the development of food allergen-specific IgG monoclonal antibodies as novel therapeutics for food allergy. Here we provide a detailed review of the immunomodulatory effects of IgE and IgG antibodies as they relate both to innate effector cell functions and to downstream regulation of adaptive immune responses in food allergy.

### IgE and Regulation of IgE Receptor Density

IgE and its receptors have symbiotic relationships. It has been known for many years that plasma IgE levels and basophil FcϵRI expression are regulated in tandem in atopic patients (109), but until recently it had not been possible to distinguish between correlation and causation. Saini and colleagues showed that the relationship between circulating IgE and basophil FcϵRI levels holds up across a spectrum of allergic, infectious, and immunodeficiency disorders associated with dysregulated IgE levels, indicating that atopy, itself, is not the driver and suggesting that IgE antibodies themselves have an effect on FcϵRI levels (110). Such a direct effect of IgE on FcϵRI expression was supported by studies of a rat basophilic leukemia line in which FcϵRI levels increased when IgE antibodies were added to the culture (111). Subsequent mouse genetic analyses showed that this interaction is indeed operative *in vivo* at physiologic IgE levels. IgE^-/-^ mice had greatly reduced FcϵRI expression both on circulating basophils and on tissue mast cells, and both cell types rapidly upregulated FcϵRI following IgE infusion (112, 113). This phenomenon could be reproduced in culture using freshly isolated primary mast cells, or cultured bone marrow derived mast cells (BMMCs), and has subsequently been confirmed in human mast cells, basophils, and dendritic cells (114–117). IgE-mediated modulation of FcϵRI levels, in turn, regulates effector cell activation thresholds. In the presence of low ambient IgE, decreased basophil surface FcϵRI is associated with an increased threshold for activation by allergen (115). The presence of IgE stabilizes FcϵRI, preventing internalization and degradation. As FcϵRI continues to be synthesized within a cell, the presence of IgE favors the capture and accumulation of FcϵRI at the cell surface (118). A similar relationship exists for IgE and CD23. Occupancy of CD23 by IgE protects its protease-sensitive sites from cleavage, and CD23 levels are low in animals lacking IgE and increase directly in relation to ambient IgE levels (56).

### IgE and Mast Cell Homeostasis

In addition to enhancing FcϵRI levels on the surface of mast cells, IgE antibodies promote mast cell survival and proliferation *in vitro* and *in vivo*. The derivation and propagation of mast cells from bone marrow stem cells require the presence of IL-3 and SCF. Without these cytokines, BMMCs undergo apoptotic cell death. However, the addition of IgE both enhances the proliferation of BMMCs in the presence of IL-3 and SCF, and protects them from apoptosis upon withdrawal of IL-3, suggesting an important role for IgE in mast cell expansion in settings of type 2 inflammation, and for survival in growth factor limiting conditions (64, 65). Such a function of promoting mast cell expansion and survival has in fact been observed in mouse models of parasitic worm infections. IgE promotes splenic mast cell expansion and parasite clearance in the course of *Trichinella spiralis* infection (119). Similarly, in an asthma model, sensitization of mice by inhalation of the fungus *Aspergillus fumigatus* drives mast cell expansion in the bronchus, trachea, and spleen and this expansion is dependent on the presence of IgE antibodies (120). Together, these findings reveal that IgE antibodies not only act to trigger mast cell degranulation and regulate FcϵRI levels, but also promote mast cell survival and expansion.

### Downregulation of IgE Receptor by Omalizumab

Omalizumab is a recombinant humanized IgG antibody that recognizes the Fc portion of IgE. Its affinity for free IgE is greater than that of IgE for its receptor so it can effectively compete for free IgE and prevent its binding to cell surface FcϵRI. However, omalizumab cannot effectively disrupt established interactions between IgE and FcϵRI. Once free IgE is captured by omalizumab, immune complexes are formed that are eventually cleared from circulation. By reducing the ambient free IgE concentration, omalizumab leads to a downregulation of FcϵRI. Allergic patients that have been treated with omalizumab have low density of FcϵRI on the surface of basophils, mast cells, and dendritic cells (114–117). Thus, more than the removal of circulating allergen-specific IgE, this secondary effect of omalizumab on FcϵRI density is critical in its mechanism of action in the treatment of hypersensitivity reactions.

### Mast Cells and Basophils as Producers of Th2 and Pro-Inflammatory Cytokines

The role of IgE and mast cells as effectors of immediate hypersensitivity in food allergy is well characterized. However, in addition to rapidly releasing the vasoactive mediators of anaphylaxis, IgE-activated mast cells serve as an important source of immunomodulatory cytokines. Cytokine production requires the activation of a transcriptional program and is therefore delayed. Thus, several hours after FcϵRI is crosslinked, as the symptoms of immediate hypersensitivity abate, IgE-activated mast cells produce a range of chemokines and cytokines important for orchestrating the influx of innate immune cells (eosinophils and basophils) and T cells important in driving type 2 inflammation (121, 122). Indeed, IL-4, the critical inducer of Th2 responses, was first reported to be produced by mast cells, in a study conducted by Marshall Plaut, Bill Paul, and colleagues (49). Mast cells are also prolific inducers of IL-6 and TNF-α, cytokines critical in the activation of APCs and induction of inflammation. Thus, mast cells, present in abundance in the gastrointestinal tract, are prime candidates to be the innate immune inducers of immunological sensitization and Th2 responses. Similar to mast cells, IgE-activated basophils are potent producers of cytokines, particularly IL-4 and, following adjuvant exposure, TSLP and IL-25 (50–52, 123).

### Mast Cells, Basophils, ILC2s, and IgE Antibodies Act as Adjuvants in Mouse Models

Upon IgE-mediated activation *via* FcϵRI, mast cells positioned at mucosal surfaces and in the skin as well as basophils recruited to these sites can prime the allergic immune response by influencing both innate and adaptive immune cells. Effective induction of T cell responses to contact sensitizers applied to the skin occurs only when mast cells and IgE are present, and local cutaneous inflammatory responses to superantigens are mast cell dependent (124, 125). Mast cell-derived TNF-α is thought to induce Langerhans migration from the epidermis to draining lymph nodes in contact sensitivity models (126) and mast cell derived histamine may activate Langerhans activation in mice injected intradermally with IgE followed by antigen challenge (127). Although basophils have not been implicated in pathways of immune priming in contact sensitivity, they have, as already noted, been shown to contribute to immunological priming in the skin in the setting of allergic inflammation induced by M903 application or barrier disruption by SDS (21, 23). When basophils are depleted or when basophils lack the ability to produce IL-4, the outcomes of sensitization and challenge are altered, with reduced allergen-induced responses (22, 23). In some models of asthma, mast cells, basophils, and IgE play important roles in orchestrating allergic sensitization and effector responses (128–130). The role of mast cells as endogenous adjuvants is most pronounced in settings where artificial adjuvants are not employed, as shown by Galli and colleagues in an alum-independent mouse model of OVA-driven asthma (128).

The constitutive presence of mast cells in the intestinal mucosa has drawn attention to their potential contribution to immunological priming in the gut in food allergy. An adjuvant role of mast cells in food allergy was demonstrated by Burton and colleagues using a mouse model of peanut allergy (131). They found that two independent strains of mice lacking mast cells, Kit^W-sh^ and Mcpt5^cre^iDTR, exhibited decreased peanut-specific IgE production and impaired peanut-specific Th2 responses, but maintained relatively robust induction of Tregs. They further established that signaling pathways downstream of FcϵRI are needed to drive allergic sensitization. For instance, mast cell lineage-specific deletion of Syk kinase in Mctp5^cre^-Syk^fl/fl^ mice, and pharmacologic inhibition of Syk kinase function, both separately recapitulated the phenotype of suppressed peanut allergy. Furthermore, blockade of IgE with anti-IgE antibodies and genetic removal of IgE (IgE^-/-^ mice) both independently led to impaired Th2 responses to peanut ingestion and still permitted the development and expansion of Tregs. The observation that mast cell-deficient mice reconstituted with wild type, but not IL-4-deficient, mast cells had restored Th2 responses to peanut strongly implicated mast cells as a key source of IL-4 in this system. IL-4 also negatively impacts the numbers and functions of Tregs so that, while promoting Th2 immunity, it also suppresses and potentially subverts the mechanisms that keep it in check. Chatila and colleagues showed that IL-4 can subvert Tregs to a pathogenic phenotype, expressing Th2 transcription factor GATA-3 as well as IL-4, a state which contributes to, rather than suppresses, allergic disease (29). Taken together these findings established the critical roles of gut mast cells as inducers of Th2 immunity, a function that, during recurrent allergen exposures and evolving adaptive immune responses, is amplified by food-specific antibodies, which signal *via* FcϵRI.

In contrast to the analyses of basophil contributions to immune sensitization in the skin, the role of basophils in priming immune sensitization to ingested allergens has been less extensively studied. Using basophil depletion (Ba103 anti-CD200R3 mAb) or basophil-deleted Bas-TRECK mice, Kawakami and colleagues demonstrated attenuation of clinical scores, diarrhea incidence and plasma levels of the mast cell protease mMCP-1 following intragastric allergen instillation in mice primed intraperitoneally and then enterally challenged with OVA (132). Total and OVA-specific IgE responses were not different between basophil-deficient and -sufficient mice arguing against adjuvant immune priming effect in this model. Along with mast cells, basophils also contribute to systemic anaphylaxis. In a mouse model of peanut induced anaphylaxis, selective, or inducible ablation of basophils, without affecting the mast cell compartment, has been shown to reduce hypothermia (133).

In addition to mast cells and basophils, ILC2s have been identified as critical innate immune sources of IL-4 and inducers of Type 2 immune responses. It turns out there is a critical interplay between these cell types, revealed in murine models of food allergy. Like Th2 cells, ILC2s express the transcription factor GATA-3 and secrete Th2 cytokines, including IL-5 and IL-13. Unlike Th2 cells, they lack T cell receptor (TCR) and cannot recognize antigen. In response to epithelial-derived cytokines, such as IL-25 and IL-33, they produce large amounts of IL-5, IL-9, and IL-13 (134). Recent findings indicate that in addition to being primed by epithelial cell-derived cytokines, ILC2s can also be activated in a mast cell driven manner, and conversely that effects of ILC2s on mast cells can influence the severity of anaphylaxis in food allergy. In mouse models of food allergy using OVA or peanut, the induction of ILC2s was significantly impaired in mice lacking IgE antibodies and those lacking mast cells (135). Furthermore, in these same murine models, IL-13 produced by ILC2s can regulate the severity of anaphylaxis by increasing sensitivity of target organs to mediators of hypersensitivity reactions (135). Recent work by Leyva-Castillo et al. in studies of food allergy-induced by epicutaneous food exposure revealed that the interaction between mast cells and ILC2s might be bidirectional. They found that intestinal mast cell expansion driven by mechanical skin injury and allergen exposure requires IL-4 and IL-13 derived from ILC2s (18).

### Regulation of Allergic Responses by IgE Antibodies in Humans and Mice With Humanized IgE Receptor Expression

The roles of mast cells and IgE in regulating Th2 responses in allergic disease in humans are not as clearly established but there is some evidence for such a connection. Testing the immunomodulatory effects of IgE blockade in OIT with food-allergic subjects offers an opportunity to test this question. In humans, omalizumab has been reported to facilitate more aggressive up-dosing, while also reducing allergic reactions during the course of the treatment (136–138). Stranks et al. hypothesized that IgE blockade might also alter the immunological changes that are induced by OIT. The authors tested this in the PRROTECT cohort of highly peanut-allergic subjects, randomized to receive either standard OIT or OIT in combination with omalizumab (139). While their analysis was somewhat limited by the fact that patients had the option to switch to open-label omalizumab part way through the study, which most did, the analysis revealed that those initially assigned to the omalizumab group, who therefore received the initial peanut dose escalation in the setting of IgE blockade, exhibited a more robust induction of anti-peanut IgG antibodies, one of the key markers of successful OIT (139). This finding suggests that in human food allergy IgE antibodies might have an immunoregulatory effect and that blockade of its receptors or their signaling pathways in mast cells might be an effective strategy for preventing or reversing food allergy. In addition to enhancing tissue-resident mast cell production of IL-4, to prime and consolidate local Th2 and IgE responses, IgE antibodies may further promote adaptive immune responses by enhancing the ability of APCs to prime T cells. Both IgE receptors, FcϵRI and CD23, are expressed by APCs and can mediate internalization of allergen complexed with IgE (140).

In humans, the trimeric form of the high affinity IgE receptor, FcϵRI, is expressed on APCs. Studies of the skin of patients affected by atopic dermatitis have revealed the presence of several FcϵRI^+^ APCs. These include Langerhans cells and inflammatory dendritic cells (which do not contain Birbeck granules) in the epidermis, and dermal dendritic cells. FcϵRI is markedly upregulated on these cell types during allergic flares. However, the lack of FcϵRI expression by murine APCs has made it challenging to investigate whether it, like CD23, might promote T cell responses *in vivo*. Mice with humanized expression of FcϵRI, using an FcϵRI α−chain transgene driven by the CD11c promoter constitutively active in APCs, have proven useful in answering this question. These animals were used to show that allergen-specific IgE, acting *via* FcϵRI on APCs, instructed naïve T cells to differentiate into Th2 cells, resulting in augmented allergen-specific Th2 responses *in vivo* (141).

### Immunoregulatory Effects of CD23

The ability of CD23 to participate in the priming of T cell responses *in vivo* was first reported in mice as facilitated antigen presentation, a process whereby IgE antibodies generated in response to a previous allergen encounter, amplify Th2 responses upon re-exposure to that allergen in a mechanism mediated by CD23 (see **Figure 2**). Recent studies of facilitated antigen presentation by Heyman and colleagues suggest that IgE:allergen complexes, bound to circulating B cells *via* CD23 enter splenic B cell follicles, where antigen is transferred to resident dendritic cells *via* B cell exosomes generated in a protease (ADAM10) and CD23-dependent process, activating them for efficient antigen presentation (142). Consistent with this model, exogenous IgE does not augment T cell responses in CD23^–/–^ mice but does enhance humoral and cellular immunity following reconstitution with CD23^+^ B cells. Both the diversity of the IgE repertoire for specific allergens (the range of recognized epitopes) and the avidity of the pooled IgE for antigen affect the efficiency of facilitated antigen presentation (143).

CD23 expressed by B cells appears to play a role in regulating IgE synthesis. Ligation of the receptor by IgE suppresses IgE production and CD23-deficient mice exhibit stronger and longer-lasting IgE responses after immunization (144–146). Conversely CD23 transgenic animals exhibit decreased IgE production (147, 148). In humans, treatment with lumiliximab, a CD23-blocking monoclonal antibody, lowers IgE levels (149). In contrast, soluble fragments of CD23 (sCD23) seem to promote IgE synthesis, perhaps by competing for IgE binding with cell-bound CD23 (see **Figure 2**) (149–151).

## The Regulatory Effects of lgG, Signaling *via* FcγRIIb IN lgE-Mediated Food Allergy

### Natural Resolution of Food Allergy Is Associated With IgG Induction

Many children with low to moderate levels of food-specific IgE antibodies can ingest the foods to which they are sensitized without exhibiting any reaction. On the one hand, this observation creates a tremendous challenge for allergy clinicians trying to establish or rule out food allergy using IgE testing. Oral food challenges, in a clinical setting, are often required to establish with certainty whether a child is tolerant or allergic. On the other hand, the inconsistent correlation between food-specific IgE and reactivity provides an important clue regarding the regulatory factors that might block immune responses to foods. There is now abundant evidence that IgG antibodies account both for natural protection from allergic reactions to foods in patients harboring food allergen-specific IgE, and that the induction of IgG responses underlies, at least in part, the protective effects of OIT. An analogous situation has been described for respiratory allergy. In large population-based cohort studies in Australia and the UK, Custovic and colleagues have established that aeroallergen-sensitized children, with aeroallergen-specific IgE antibodies, often have no symptoms. This led to the concept of “benign Th2 immunity” and further analysis of these subjects revealed that those with higher allergen-specific IgG/IgE ratios had fewer symptoms and their sera inhibited the activation of basophils sensitized with aeroallergen-specific IgE (152, 153). One challenge in diagnosis is that circulating IgE and IgG levels may not reflect the amounts of these antibodies present in the mucosal tissues where allergic reactions are initiated.

Several investigations have revealed that natural resolution of milk allergy in children is associated with increasing IgG levels (154, 155). Similarly, among subjects who test positive for IgE antibodies to peanut, Santos and colleagues found that higher levels of specific IgG4 correlate with tolerance (156). For the most part, analyses of any protective effects of IgG in food allergy have focused on the IgG4 isotype. A protective role for IgG4 had previously been established to be a very strong biomarker of efficacy in subcutaneous allergen immunotherapy (SCIT) (157). The IgG4 findings in SCIT led to a focus on this subclass in food allergy studies and it is the only subclass of IgG for which specific tests have been commercially developed. IgG4 is the least abundant isotype of IgG in human serum, often representing only about 5% of total IgG (158, 159). However, with chronic antigen exposure, IgG4 can increase to account for a larger fraction of total IgG (160). Like IgE, IgG4 is induced during Th2 immune responses under the action of IL-4 and IL-13 on B cells. The concept of a “modified Th2 response,” has evolved to describe a scenario where IL-10 is present along with IL-4, and IgG4 class switching and production is promoted over IgE (159, 161–163).

IgG4 is the sole subclass of IgG in that it does not mediate common IgG effector functions such as antibody-dependent cell-mediated cytotoxicity or complement dependent-cytotoxicity. IgG4 antibodies also exhibit a unique ability to undergo Fab arm exchange (FAE). In this process, heavy chains of IgG4 antibodies can separate into half antibodies, each consisting of one heavy chain and one light chain. Half antibodies originating from different parent IgG4s can combine to form bispecific antibodies (164). This re-assortment of Fab regions potentially allows a single IgG4 to recognize two epitopes on an allergen, both increasing overall binding avidity and facilitating crosslinking. Due to its presence in the serum at higher concentration than IgE, its ability to recognize more epitopes and its limited ability to form immune complexes and mediate effector function, it has been proposed that IgG4 might be uniquely suited to function as a blocking antibody, intercepting allergens before they can be engaged by FcϵRI-bound IgE on the surface of effector cells.

### Induction of High Levels of Specific IgG Following OIT

Perhaps the most compelling evidence for a role of IgG antibodies in regulating food allergen responsiveness has come from OIT studies. In OIT, an allergenic food is administered orally in daily doses that incrementally increase, often up-dosing weekly, over the course of several months. Upon completion of OIT, patients can typically tolerate significant amounts of the allergen without reaction. As this acquired ability to ingest the food is temporary and Tregs which maintain immunological tolerance at the T cell level have not been implicated, this state is referred to as food “unresponsiveness” rather than tolerance (165). Maintenance of this unresponsive status requires ongoing ingestion of the allergenic food. It is striking that at the completion of OIT patients still have very high levels of food allergen-specific IgE antibodies, despite their ability to ingest the allergen without incident. In fact, the IgE titers typically seen after OIT can be unchanged from pre-OIT levels, and are of a magnitude that would strongly predict a significant reaction if obtained in a patient who had not undergone OIT (166). These observations are highly suggestive that OIT induces a suppressive factor, one that inhibits IgE-mediated anaphylaxis.

A clear clue as to the identity of the suppressive factor has been provided by the highly consistent observation that OIT of foods, including milk, egg, and peanut, induces strong food allergen-specific IgG4 responses (167–172). The success of baked-egg challenge has been correlated with these increases in egg-protein IgG4 following OIT (173). In contrast to prior observations with SCIT, the IgG response in OIT encompasses all IgG subclasses, not just IgG4. For instance, patients undergoing OIT for peanut allergy in the PRROTECT trial had several log increases in levels of peanut-specific IgG1, IgG2, IgG3, IgG4, and IgA, as well as the ratio of peanut-specific IgG4/IgE with the greatest increases evident in peanut-specific IgG2 and IgG4 which were increased by two logs (139, 174). The groups of Galli and Nadeau reported that robust IgG responses and elevated IgG4/IgE ratios correlate with sustained unresponsiveness following peanut OIT (175). When modeled in mice with established peanut allergy, OIT also induces a strong IgG response, inclusive of all the murine IgG subclasses (174).

### FcγRIIb-Mediated Suppression of IgE-Mediated Mast Cell Activation by Food-Specific IgG Induced During OIT

Mechanisms of OIT and the inhibitory effects of IgG have been studied both in mouse models and in mechanistic investigations in human clinical trials. Since BMMCs can be easily cultured using IL-3 and SCF, and they can be sensitized with food allergen-specific IgE, they provide an excellent tool with which to interrogate the serum of OIT-treated mice for any potential inhibitory activity of induced IgG. Surface expression of the granule marker LAMP-1 (CD107a), which is extruded upon degranulation, is a sensitive marker of mast cell activation. Within minutes of exposure to allergen, mast cells degranulate and an increase in LAMP-1 on the cell surface can be detected. Burton and colleagues reported that sera from mice that underwent OIT for peanut allergy can inhibit the peanut-induced activation of BMMCs sensitized with specific IgE in an IgG-dependent manner (174). In addition to inhibiting IgE-induced degranulation, OIT-induced food allergen-specific IgG antibodies also inhibit the production of IL-4 and IL-13 by activated BMMCs (176). Under these conditions, suppression of IgE activation of IgG is not observed in FcγRIIb^-/-^ BMMCs, indicating a requirement for this receptor in IgG-mediated inhibition. At high doses of IgG, however, inhibition of mast cell activation can be exerted even in cells lacking FcγRIIb, indicating that, in addition to sending negative signals *via* FcγRIIb, IgG can also act as a blocking antibody, sterically preventing the interaction of allergen with FcϵRI-bound IgE (see **Figure 1**) (174). Prior to the identification of the key role of FcγRIIb in mediating the suppressive actions of IgG on mast cells and basophils, this steric blocking effect had commonly been presumed to be the dominant inhibitory mechanism of IgG in allergy, especially following SCIT, which is why such IgG antibodies are still commonly referred to as blocking IgG.

The physiologic relevance of IgG : FcγRIIb-mediated inhibition of IgE-triggered mast cell activation to allergic reactions *in vivo*, particularly to anaphylaxis, has been demonstrated using mouse models. FcγRIIb-deficient mice have provided an excellent genetic tool to analyze this biology. Sensitized FcγRIIb-deficient mice exhibit enhanced anaphylaxis upon allergen challenge (176). Furthermore, inhibition of anaphylaxis by passive transfer of allergen-specific IgG occurs in wild type but not FcγRIIb-deficient mice, indicating that restoration of tolerance *via* IgG requires IgG : FcγRIIb interactions. Although FcγRIIb^-/-^ mice lack the receptor on all cells that would normally express the receptor, reconstitution of the mice with cultured FcγRIIb^+/+^ mast cells from wild type donors restores the protective effects exerted by passive administration of exogenous allergen-specific IgG, confirming the central role of mast cell FcγRIIb in regulating IgE-mediated anaphylaxis *in vivo* (see **Figure 1**) (176).

### Inhibition of Basophil Activation by Post-OIT Serum in Human Subjects

**Figure 3:**
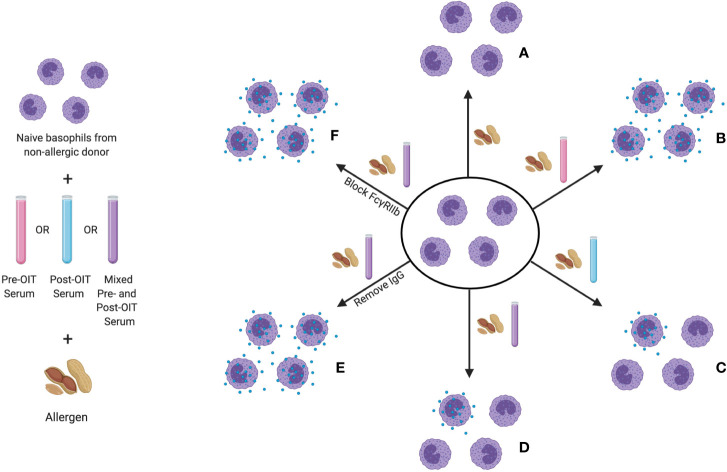
The indirect basophil activation test (iBAT) as a probe for inhibitory food allergen specific IgG. In this assay, basophils from a non-allergic donor are sensitized with IgE from an allergic donor, then incubated with serum to be queried (typically pre-OIT serum, post-OIT serum, or a mix of the two) and then exposed to allergen. Conditions assayed include (proceeding clockwise starting from top center): **(A)** basophil incubation with allergen in the absence of serum (no activation), **(B)** with serum from an allergic donor (full degranulation), **(C)** with post-OIT serum (suppressed activation), **(D)** with a mix of pre- and post-OIT serum (suppressed activation if inhibitory activity is present), **(E)** post-OIT serum with IgG removed (to query the contribution of IgG to suppression) and **(F)** post-OIT serum with antibodies to FcγRIIb (to test whether inhibition is receptor-mediated).

The same receptor-mediated inhibition of IgE-induced activation by IgG has also now been clearly demonstrated in patients undergoing OIT. Using an indirect basophil activation test (iBAT), Burton and colleagues analyzed suppressive activity in the serum of peanut-allergic patients who underwent OIT (174). In the iBAT, basophils from a non-allergic donor are used to interrogate the sera of study subjects for both activating and suppressive factors (see **Figure 3**). IgE-containing serum, typically from a peanut allergic patient, is added to these basophils in culture. After the addition of peanut extract, granule extrusion is measured by flow cytometric quantitation of CD63, a granule protein that is similar to the LAMP-1 marker used in the murine system. Using this assay, the group found decreased basophil activation by iBAT following completion of OIT. When both pre- and post-OIT serum from the same individual were incubated with donor basophils, the degranulation was lower than that induced by pre-OIT sera alone, indicating that the suppressive activity was OIT-induced (176). It was determined that this suppression is IgG-mediated and could be blocked by antibodies to FcγRIIb.

Similar observations were subsequently reported by Santos et al. in a separate peanut-allergic cohort. Rather than using basophils from non-allergic donors to query the OIT sera, this group used the human mast cell line, LAD2 (156). They too observed IgG-mediated suppressive activity in LAD2 activation by post-OIT sera, and found that specific depletion of IgG4 reduced the suppression leading them to the conclusion that post-OIT suppression is IgG4-mediated. In reviewing the Santos report, however, it is important to note that while IgG4 removal reduced the degree of suppression exerted by post-OIT sera, the effect was incomplete. IgG4-depleted sera from post-OIT subjects in their cohort exerted >50% suppression of basophil activation compared with 80% in sham-depleted sera, which actually suggests that most of the suppressive activity might in fact be accounted for by non-IgG4 isotypes (156). At this point the argument could be made that suppression of FcϵRI signaling by FcγRIIb in food allergy is not uniquely accounted for by IgG4, but is also exerted by other IgG subclasses, all of which are known to have measurable affinity for FcγRIIb. Like OIT, serum from patients that had undergone SLIT can also inhibit basophil reactivity in an IgG dependent manner (177). Future studies with monoclonal IgG antibodies, expressed as IgG isotype swap variants, are needed to clearly delineate the potential contributions of these isotypes to FcγRIIb-mediated basophil suppression.

The discovery that food-specific IgG antibodies account, at least in part, for suppression of food reactions in IgE^+^ food-tolerant subjects and those who have completed OIT has seeded interest in potential therapeutic applications of IgG in food allergy. Although inhibitory food-specific IgG antibodies are still in development by pharmaceutical companies, and there are not yet any direct data regarding the efficacy of IgG in preventing food anaphylaxis, there are preclinical studies that support the approach. Burton and colleagues recently developed a humanized mouse model in which NOD-scid IL2Rγ^null^ mice (NSG,NOD.Cg-Prkdc^scid^ Il2rg^tm1Wjl^/SzJ), given human CD34^+^ stem cells, exhibit robust T cell expansion, including Foxp3^+^CD25^+^ Tregs, IFNγ^+^ Th1 cells and IL-4^+^ Th2 cells, and B cell engraftment (178). These animals are readily sensitized to ingested peanut, producing specific IgE and exhibiting anaphylaxis with elevated plasma tryptase levels upon challenge (178). If post-OIT serum is administered 24 h prior to allergen challenge, the mice are protected from peanut-induced anaphylaxis (179). This IgG-mediated inhibition can be blocked by injecting mice with anti-FcγRIIb (179). However, blocking FcγRIIb does not fully restore the anaphylaxis phenotype, suggesting that IgG is able to exert some inhibition in a manner independent of FcγRIIb, most likely through steric hinderance (179).

### Tissue-Specific Variations in FcγRIIb Expression

The expression of FcgRIIb on human mast cells is still an active area of investigation including the factors that regulate the expression of the receptor. Comparison of the expression of FcγRIIb on mast cells residing in various human tissues has revealed that FcγRIIb is absent from dermal mast cells, but is expressed on those in the gastrointestinal tract (179, 180). This is recapitulated in humanized mice, in which FcγRIIb is detectable by qPCR and flow cytometry in the small intestine and spleen, but not in the skin (179). As would be anticipated by the lack of FcγRIIb, *in vitro* IgG-mediated inhibition of mast cell degranulation does not manifest in human skin mast cells. This low, or completely absent, expression of FcγRIIb on dermal mast cells likely explains why patients who have successfully undergone desensitization therapy are able to consume the allergen without symptoms, but they may still exhibit positive skin prick test responses to allergen (181–184).

The relevance of FcγRIIb to physiologic regulation of allergic responses is further suggested by genetic associations. An asthma family cohort study that included 370 atopic, 239 non-atopic, and 169 asthmatic subjects, identified a functional SNP in FCGR2B (187Ile>Thr) that was associated with atopy and IgE production (185). Functional analysis of this variation, that is located in the transmembrane segment of the receptor, showed that 187Ile>Thr FCGR2B is less effective at mediating inhibitory signals than the common allele (186–188).

### IgG-Mediated Inhibition of Sensitization to Ingested Antigens

**Figure 4:**
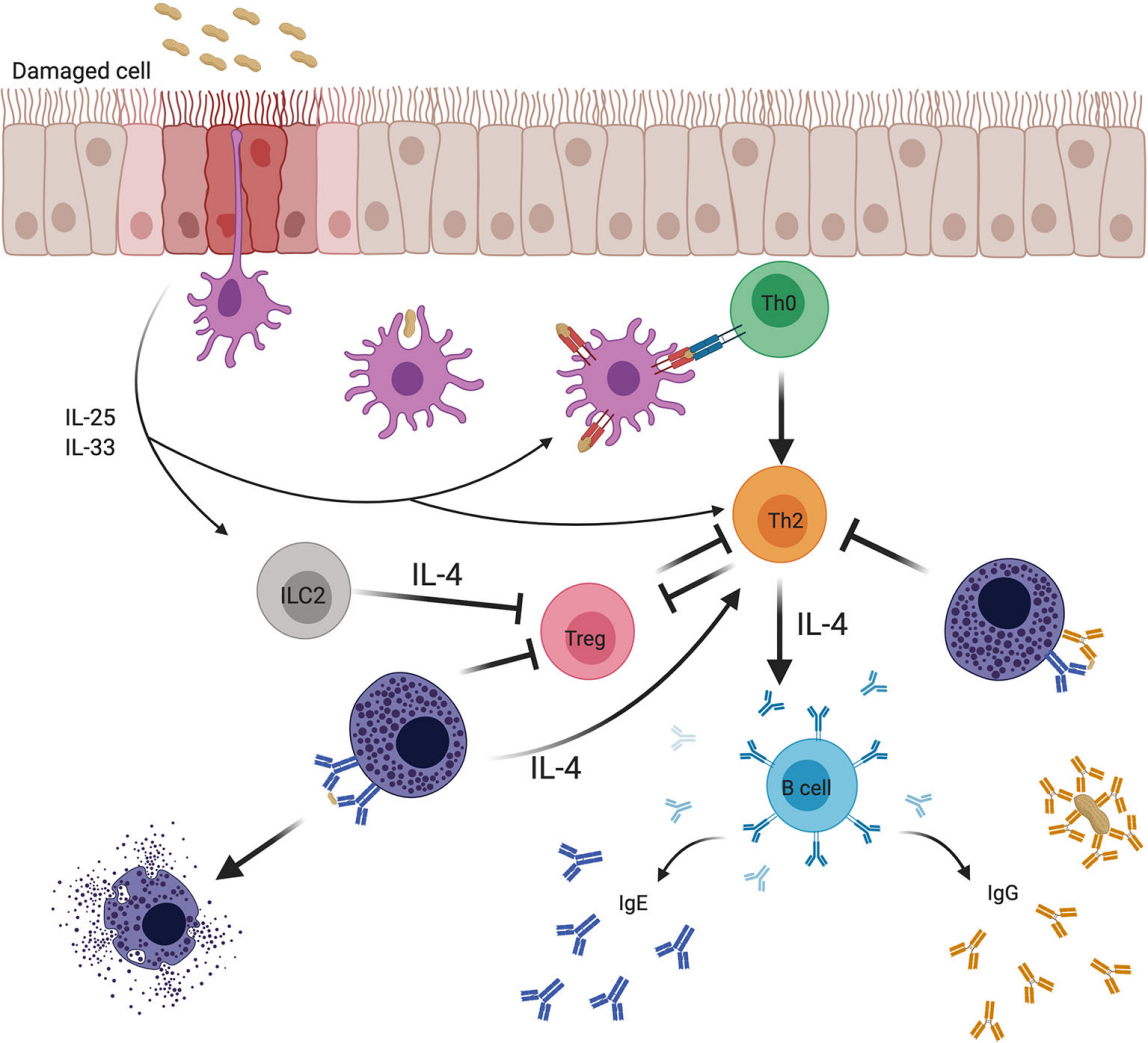
Effects of mast cells on adaptive immune responses to food allergens and the regulation of these effects by IgE and IgG antibodies. Food antigens pass through damaged epithelium, specialized intraepithelial passages (8), or are sampled by antigen presenting cells (APCs). Epithelial cells subjected to stress or microbial signals secrete cytokines such as IL-25 and IL-33 that promote the activity of various cellular mediators involved in the breakdown of tolerance. Mucosal APCs present antigen to naïve T cells that mature into Th2 cells in the context of a Th2-conducive environment. Th2 cells are known to both depend on IL-4 for their differentiation and survival, and to produce IL-4 that drives IgE isotype switching by B cells and mast cell expansion, while inhibiting the production of regulatory T cells (Tregs) and subverting their function. Mast cells sensitized with allergen-specific IgE and type 2 innate lymphoid cells (ILC2s) provide a priming source of IL-4, initiating and sustaining the Th2 environment. In contrast, allergen-specific IgG antibodies induced during natural allergen resolution or during OIT can inhibit mast cell activation *via* signaling through FcγRIIb receptor. Inhibition of mast cell activation by IgG can break the positive feedback loop between mast cells and Th2.

As one might predict, based on their ability to prevent IgE-induced production of Th2 cytokines by mast cells in culture, IgG antibodies that are administered prophylactically prior to initial allergen ingestion can hinder the development of Th2 responses and IgE antibodies. This possibility was assessed in a recent investigation using mouse model of food allergy involving repeated administration of OVA in *Il4raF709* mice, a strain rendered inherently atopic by knock-in of a variant IL-4R α-chain (176). Administration of allergen-specific, but not control, IgG during the sensitization phase markedly suppressed production of OVA-specific Th2 cells, and was permissive for the expansion of Tregs which was not observed in controls (176). Allergen-driven mast cell expansion was also suppressed in the IgG-treated mice. As would be expected in the setting of decreased Th2 immunity, IgE responses were suppressed by more than one log. The combination of low IgE and decreased mast cell numbers rendered the OVA IgG-treated animals completely resistant to anaphylaxis, with no signs of hypothermia, a cardinal physiologic feature of anaphylaxis in mice, or elevated levels of plasma mast cell protease-1 (MMCP-1), a granule protease and marker of mast cell activation analogous to tryptase in humans. Taken together, these findings show that, in addition to blocking phenotypes of IgE-mediated immediate hypersensitivity, like anaphylaxis in the setting of established food allergy, IgG antibodies can block the development of food allergy by blunting the Th2 adjuvant function of mast cells (see **Figure 4**). Analysis of FcγRIIb^-/-^ mice in the same study revealed a key role for the inhibitory receptor in mediating the protective effect of IgG.

In a study of fish allergy, active immune induction of IgG antibodies by vaccinating mice with a hypoallergenic mutant of the fish allergen *Cyp c 1*, has also been shown to protect against allergy (189). These findings suggest that an IgG-based preventive strategy might be beneficial as a prophylactic treatment for children at risk for developing food allergies. The recent findings of Oyoshi and colleagues from studies done in mice, in which IgG:allergen immune complexes passed by food allergen-tolerant mothers to their offspring *via* breast milk, support the induction of Tregs and suppress IgE responses, implicate IgG in physiologic maternal transfer of tolerance (190). Furthermore, offspring of mothers who have high levels of allergen-specific IgG in their plasma, cord blood, and breast milk, have lower incidence of allergen sensitization (191). However, the role of mast cells and FcγRIIb in mediating this tolerogenic mechanism have not yet been explored.

### Restoration of Tolerance During Adjunctive Therapy With IgG During OIT

OIT can also be modeled in OVA-sensitized *Il4raF709* mice. Mouse models of food allergy using this line have been applied to test the hypothesis that allergen-specific IgG given during the course of OIT might enhance the effectiveness of OIT, by dampening Th2-inducing signals. As expected, OIT, even without adjunctive IgG, resulted in diminished allergen sensitivity, as assessed by anaphylaxis (hypothermia) and MMCP-1 release. However, the protective effects of IgG were dramatically amplified in mice receiving OVA-specific IgG during their OIT with complete abrogation of anaphylaxis and markedly blunted Th2 and IgE responses (176).

### IgG Antibodies in Allergic Diarrhea

IgE-mediated gastrointestinal reactions, including diarrhea, are common in IgE-mediated food allergy. The effects of food allergen-specific IgG antibodies in both anaphylaxis and diarrhea were investigated by Kucuk and colleagues in a hybrid model of food allergy involving both active sensitization and passive transfer of IgG (192). Though anaphylaxis and diarrhea are both IgE- and FcϵRI-mediated, the mast cell mediators driving these phenotypes appear to be different. Histamine is associated with anaphylactic shock, while platelet activating factor and serotonin are associated with diarrhea (86, 193). To address whether IgG can confer protection against the diarrhea phenotype, Kucuk et al. tested if anti-TNP IgG1 administration prevents the diarrhea induced by TNP-BSA challenge in sensitized mice. When the investigators tested the phenotypes on the FcγRIIb^-/-^ background, protection against anaphylaxis by IgG1 antibodies was no longer observed while IgG1-mediated inhibition of diarrhea was, surprisingly, retained (192). These findings suggest that while IgG-mediated inhibition of shock is dependent on signaling *via* FcγRIIb as has been confirmed by others, that inhibition of diarrhea may be due to steric hinderance of antigen-IgE binding rather than a receptor-mediated mechanism (see **Figure 1**).

### Allergen-Specific IgG and IgE Repertoire Overlap and the Role of IgG^+^ B Cells as Custodians of IgE Memory

For optimal choreography of allergen-specific interactions between IgE and IgG antibodies in activating or suppressing food allergen responses one might predict that overlap of their repertoires would be advantageous. Recent findings regarding the relationship between IgE and IgG memory suggest that such coordination exists. The process of B cell isotype class switching to IgG^+^ from IgM^+^ precursors as well as the evolution of high affinity IgG responses to antigens through affinity maturation, and the creation of memory B cell clones all occur in germinal centers of lymph nodes. It turns out that, in contrast to IgG^+^ B cells, mouse germinal center IgE^+^ B cells are susceptible to apoptosis and tend to rapidly transition to a CD138^+^ plasmablast phenotype. An analysis of sorted human IgE^+^ B cells from allergic subjects by Croote et al. *via* single cell sequencing revealed that they, like their murine equivalents, almost all have plasmablast transcriptional signatures (194). This unique fate of IgE^+^ B cells may be related to their very low surface levels of IgE compared with surface IgG or surface IgM on B cells expressing those isotypes (195–198). Both IgE affinity maturation and memory seem to require an intermediate IgG^+^ stage during which this process occurs followed, sequentially, by a second isotype switch from IgG^+^ to IgE^+^. The presence of hybrid switch sequences, Sµ-Sγ-Sϵ in many IgE^+^ B cells serves as a footprint of their previous existence as IgG^+^ clones. The requirement for the intermediate IgG stage in affinity maturation is demonstrated by the lack of high-affinity IgE responses in mice lacking the Cγ locus (199). Deep sequencing of millions of peripheral blood IgH genes in allergic subjects by Boyd and colleagues revealed a phylogenetic lineage progression in which all somatically-mutated IgE sequences were derived from identically-mutated IgG parent clones (200). These findings are consistent with a mechanism in which IgE^+^ and IgG^+^ B cells have a shared allergen specific repertoire, with memory residing in the IgG^+^ population.

Furthermore, T follicular helper (Tfh) cells are required for antibody isotype switching by B cells (201, 202). IL4 producing T follicular helper (Tfh) cells are needed for IgE production and IgE production can be limited by regulatory T follicular (Tfr) cells (203). Eisenbarth and colleagues have recently described a specific subset of IL-4- and IL-13-producing Tfh cells that can drive the production of IgE with high affinity to the antigen (204).

### Therapeutic Applications of IgG

Harnessing the evolving understanding of the importance of allergen specific IgG in regulating both immediate hypersensitivity and chronic type 2 responses in allergy, researchers have developed recombinant allergen specific IgG antibodies or molecules that bind FcγRIIb and could be used to treat IgE-mediated allergies. In a recent clinical trial in cat-allergic subjects, Orengo et al. reported that administration of a single high dose of a pair of *Fel d 1*-specific monoclonal IgG4 antibodies in humans prevented symptoms following cat allergen exposure. The dose of IgG4 results in levels in plasma that are in vast excess of the circulating IgE, comparable to the IgG levels induced during successful immunotherapy. In as little as 8 days following injection of *Fel d 1* specific IgG4, subjects challenged with cat allergen had a decrease in total nasal symptom score and an increase in peak inspiratory flow compared to controls. By day 29, their skin prick test reactivity was decreased (205). As animal studies, discussed above, show that allergen-specific IgG exerts immunomodulatory effects, skewing the T cell compartment away from a pro-inflammatory Th2 profile, it would be interesting to see if the protective effects of allergen specific humanized IgG antibodies extend beyond immediate hypersensitivity and whether these monoclonal antibodies might be valuable adjuncts for SCIT in subjects with respiratory allergy (176). Given the strong induction of allergen-specific IgG antibodies in food-allergic subjects undergoing OIT and the clear immunomodulatory effects of these antibodies, we anticipate that food-specific monoclonal antibodies currently in development will also prove beneficial.

A significant limitation of the IgG antibody approach is that any given allergen-specific IgG therapeutic would only target a single allergen. It is unclear if IgG specific for each component allergen would be required for effective therapy. Since the inhibitory signal delivered to the mast cell by IgG against any protein within a food, it is possible that targeting just one component would be sufficient. A variety of alternative approaches have been explored using bispecific antibodies or fusion proteins that bring bridge FcϵRI and FcγRIIb (206, 207). Several groups have reported the development of bifunctional FcϵRI crosslinkers composed of the Fc portion of IgG1 and the Fc portion of IgE or an allergen (208, 209). However, molecules containing the Fc portion of IgE might be limited in their activity by the availably of free FcϵRI sites. Tam et al. designed a bispecific antibody consisting of a Fab’ fragment that recognizes human IgE and a Fab’ fragment that recognizes FcγRIIb. Incubation of IgE-sensitized cord blood derived mast cells and basophils with the bispecific antibody blocked histamine release following antigen challenge (210). Similarly, Jackman and colleagues described a bispecific antibody in which one arm recognizes FcϵRI at a site not blocked by IgE binding, while the other arm binds to FcγRIIb (211).

While these molecules are interesting because of their potential to broadly inhibit allergic responses, they have several limitations. Chronic administration of these compounds is made challenging by their immunogenicity and their short half-life *in vivo*. Furthermore, experience has shown that even a very low clinical risk of IgE receptor cross linking and anaphylaxis with molecules bearing an FcϵRI-binding moiety can pose very significant barriers to their clinical advancement.

## Discussion

The quest to understand why some individuals with allergen-specific IgE experience life-threatening reactions, while others have no symptoms at all following ingestion has led to the discovery of allergen-specific IgG as the serum factor that is responsible for conferring protection against allergic reactions. Food allergen-specific IgG levels are higher in individuals that are sensitized but unresponsive to the allergen, in those who have outgrown their food allergies and in subjects who have acquired food unresponsiveness after successful completion of OIT. Furthermore, research in animal models has convincingly demonstrated that the presence of allergen specific IgG during sensitization can inhibit the production of allergen specific IgE, and subsequent promotion of Th2 immunity, while promoting effective induction of Tregs. While not studied in animals, IgE and IgG antibodies may be important to sustain food allergy in a maintenance phase, in addition to induction. Similarly, in these models, an adjunctive therapy with IgG during OIT also impairs Th2 responses and promotes the development of Tregs (176).

The factors driving IgE and IgG responses to ingested antigens are complex. Outside the scope of this review but critically important to shaping immune responses in the gut is the microbiome. Mice with reduced numbers of intestinal microbes or diminished microbial diversity (germ free mice or antibiotic-fed mice) have increased susceptibility to sensitization and food allergy (212, 213). Germ free mice exhibit higher baseline IL-33 expression in their small intestine and also greater IgE levels in their serum (9, 129). Certain species of bacteria, including some in the genus *Clostridia*, have been shown to confer protection against development of food allergies by contributing to the development of peripherally expanded protective RORγ^+^ T cells in a MYD88-dependent manner (9, 11, 213).

The specific contribution of IgG4 relative to other IgG isotypes in the inhibition of IgE-mediated effector cell activation *via* FcϵRI and in exerting the immunomodulatory effects of IgG requires further study. We believe that investigations in the field have been skewed by the exclusive availability of IgG4 reagents in the ImmunoCAP platform used by most clinical investigators. This has prevented consideration and analysis of the other isotypes. However, as noted in this review, all food-specific antibodies of all four IgG isotypes are induced during OIT and binding to FcγRIIb is clearly not specific to IgG4, begging the question of whether IgG1-3 contribute.

As presented in this review, data correlating allergen-specific IgG with protection against food allergy phenotypes and the role of FcγRIIb in blocking mast cell and basophil activation has been repeatedly shown by many groups. Though the antibody and the receptor can work together to block the activity of effector cells, research has also shown that they are also capable of exerting inhibitory effects independent of one another *via* a number of ways. These observations have been harnessed to engineer antibodies against specific allergen epitopes, and to design small molecules that can aggregate FcγRIIb to FcϵRI-bound IgE bound to antigen. As our understanding of the roles of IgG antibodies both in preventing IgE-triggered anaphylaxis and in regulating Th2 immune responses continues to evolve and as approaches for engineering both allergen-specific and broad-spectrum therapeutics are further developed, we are optimistic that effective strategies will emerge for prevention and treatment in the worldwide problem of food allergies.

## Author Contributions

Under the supervision of HCO, the manuscript was written by CK with YSEA and OLL. All authors contributed to the article and approved the submitted version.

## Funding

This work was supported by NIH grant 1R01AI119918 (HO). CK is supported by a postdoctoral fellowship from the Fonds de recherche du Québec—Santé (258617 and 285834). All figures were created with BioRender.com.

## Conflict of Interest

The authors declare that the research was conducted in the absence of any commercial or financial relationships that could be construed as a potential conflict of interest.

## Abbreviations

Mouse names: IgE knockout, *Igh-7^-/-^*; *Kit^W-sh^* mice, B6.Cg-*Kit^W-sh^*/HNihrJaeBsmGlliJ; Mcpt5cre iDTR, B6.Tg(Mcpt5^cre^)*Gt(ROSA)26Sor^tm1(HBEGF)Awai^*.
